# Cryoballoon left atrial posterior wall isolation using the cross-over technique in situs inversus with dextrocardia: a case report

**DOI:** 10.1093/ehjcr/ytag674

**Published:** 2026-09-07

**Authors:** Fuminori Odagiri, Takashi Tokano, Tetsuro Miyazaki, Kai Ishii, Tohru Minamino

**Affiliations:** Department of Cardiology, Juntendo University Urayasu Hospital, 2-1-1 Tomioka, Urayasu-shi, Chiba 279-0021, Japan; Department of Cardiology, Juntendo University Urayasu Hospital, 2-1-1 Tomioka, Urayasu-shi, Chiba 279-0021, Japan; Department of Cardiology, Juntendo University Urayasu Hospital, 2-1-1 Tomioka, Urayasu-shi, Chiba 279-0021, Japan; Department of Cardiology, Juntendo University Urayasu Hospital, 2-1-1 Tomioka, Urayasu-shi, Chiba 279-0021, Japan; Department of Cardiovascular Biology and Medicine, Juntendo University Graduate School of Medicine, 2-1-1 Hongo, Bunkyo-ku, Tokyo 113-8421, Japan

**Keywords:** Dextrocardia, Situs inversus, Cryoballoon ablation, Left atrial posterior wall isolation, Cross-over technique, Atrial fibrillation, Case report

## Abstract

**Background:**

Situs inversus with dextrocardia (SID) is a rare congenital condition with fully mirrored cardiac anatomy, posing unique technical challenges for catheter ablation of atrial fibrillation (AF). Although cryoballoon (CB)-based left atrial posterior wall isolation (LAPWI) has recently been reported in a patient with SID, adjunctive radiofrequency catheter ablation was required to complete posterior wall isolation. We present a case in which complete LAPWI was achieved exclusively using CB applications, employing the previously described cross-over technique.

**Case summary:**

A 69-year-old woman with SID and drug-refractory persistent AF underwent catheter ablation using a fourth-generation CB system. To accommodate mirrored anatomy, the procedure was performed using mirror-image fluoroscopy and intentionally reversed catheter manipulation to restore conventional spatial orientation. Pulmonary vein isolation was achieved, followed by LAPWI using the cross-over technique, in which overlapping CB applications were delivered from both left- and right-sided pulmonary vein anchor positions to improve lesion continuity across the left atrial posterior wall. Complete LAPWI was confirmed by voltage mapping and pacing manoeuvres demonstrating both entrance and exit block. No periprocedural complications occurred. The patient remained free from AF recurrence for 48 months without antiarrhythmic therapy.

**Discussion:**

This case demonstrates that complete CB-based LAPWI using the cross-over technique is feasible even in anatomically mirrored hearts. The successful use of mirror-image fluoroscopy and intentionally reversed catheter manipulation provides practical technical insights and highlights the importance of deliberate spatial adaptation when performing complex left atrial ablation in patients with SID.

Learning points

- Mirror-image fluoroscopy and intentionally reversed catheter manipulation facilitate safe and intuitive catheter ablation in situs inversus with dextrocardia.
- The cross-over technique enables contiguous cryoballoon lesion delivery across the left atrial posterior wall from both left- and right-sided pulmonary vein anchor positions.
- Complete left atrial posterior wall isolation using cryoballoon ablation alone is feasible even in anatomically mirrored hearts.

## Introduction

Situs inversus with dextrocardia (SID) is a rare congenital anomaly with an estimated prevalence of 1–2 per 20 000 in the general population.^1^ Most patients with SID do not have clinically significant structural heart disease and have a near-normal life expectancy. As this population ages, atrial fibrillation (AF) is increasingly observed. However, catheter ablation (CA) in patients with SID poses technical challenges due to mirrored cardiac anatomy and altered pulmonary vein (PV) orientation.

Although CA for AF in patients with SID has been reported using multiple energy sources,^2–6^ complete left atrial posterior wall isolation (LAPWI) using cryoballoon ablation (CBA) alone remains rarely described. We present a case in which durable LAPWI was successfully achieved using CBA alone with the previously described cross-over technique, without adjunctive radiofrequency (RF) CA.^7^

## Summary figure

**Figure:**
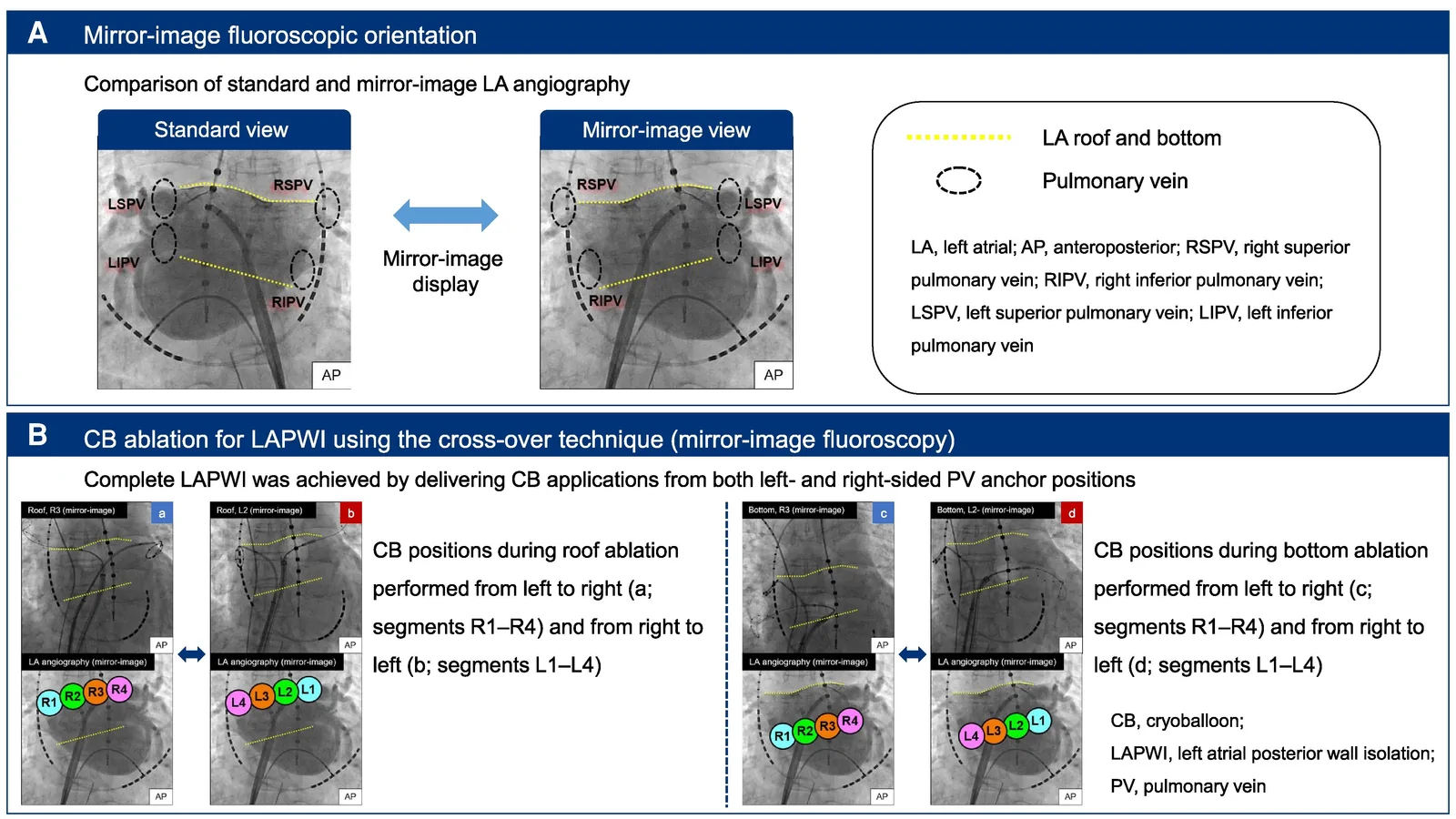
Mirror-image fluoroscopic orientation and cryoballoon (CB) left atrial posterior wall isolation (LAPWI) using the cross-over technique in a patient with situs inversus and dextrocardia. (*A*) Comparison of standard and mirror-image left atrial (LA) angiography demonstrating the mirror-image fluoroscopic orientation used during the procedure. Yellow dotted lines indicate the LA roof and bottom. (*B*) Representative fluoroscopic images demonstrating the cross-over CB technique for LAPWI. CB applications were delivered from both left- and right-sided pulmonary vein (PV) anchor positions to achieve complete LAPWI. Panels a and b show roof ablation performed from left to right and from right to left, respectively. (*C* and *D*) Bottom ablation performed from left to right and from right to left, respectively.

## Case presentation

**Figure 1:**
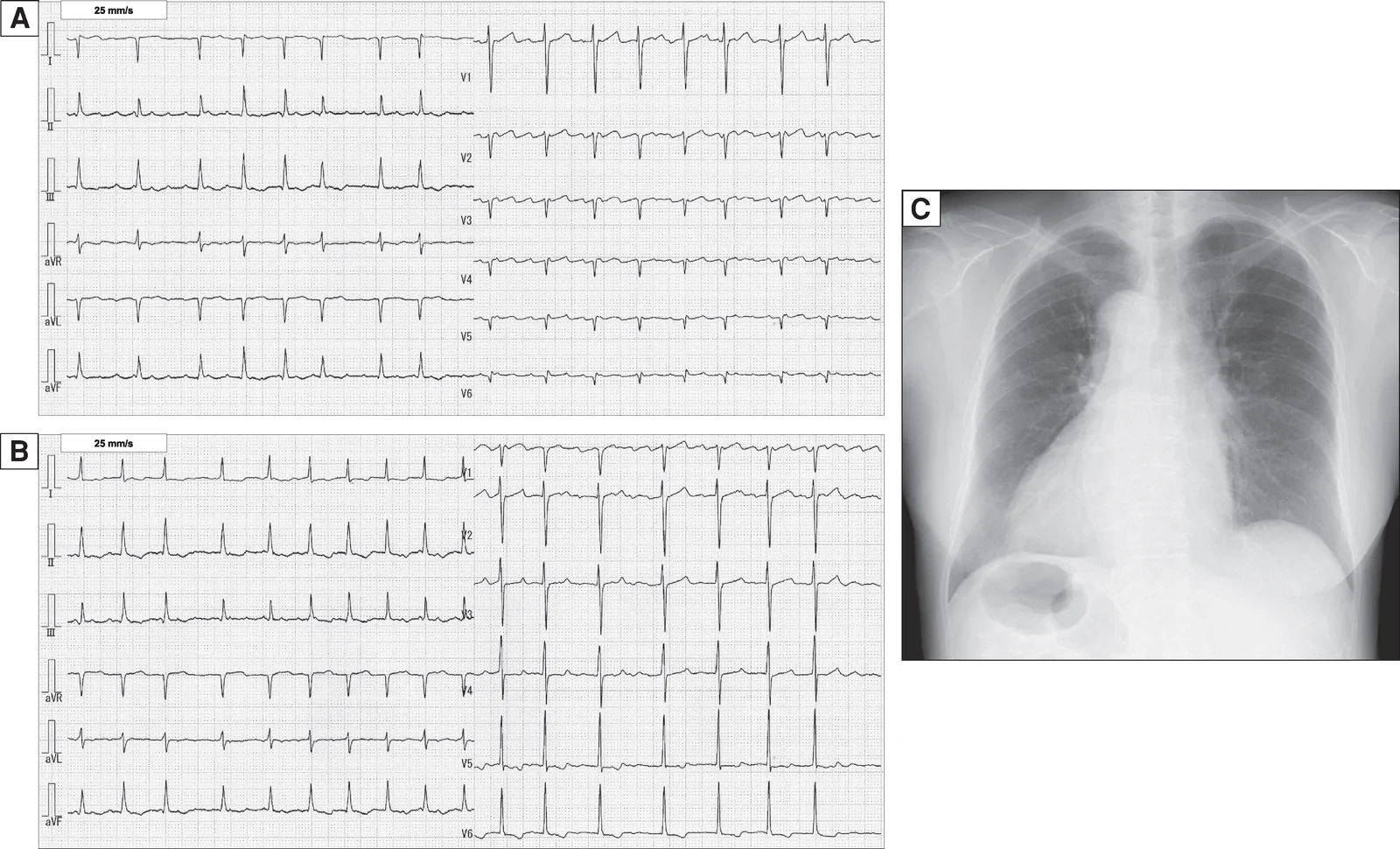
Preprocedural electrocardiogram (ECG) and chest radiograph. (*A*) ECG recorded with standard limb and precordial lead placement, demonstrating atrial fibrillation with a moderate ventricular response (98 bpm). (*B*) ECG recorded with reversed limb leads and mirror-image precordial lead placement, which provides a more conventional ECG morphology and facilitates interpretation in dextrocardia. (*C*) Chest radiograph demonstrating dextrocardia and a gastric bubble beneath the right hemidiaphragm.

**Figure 2:**
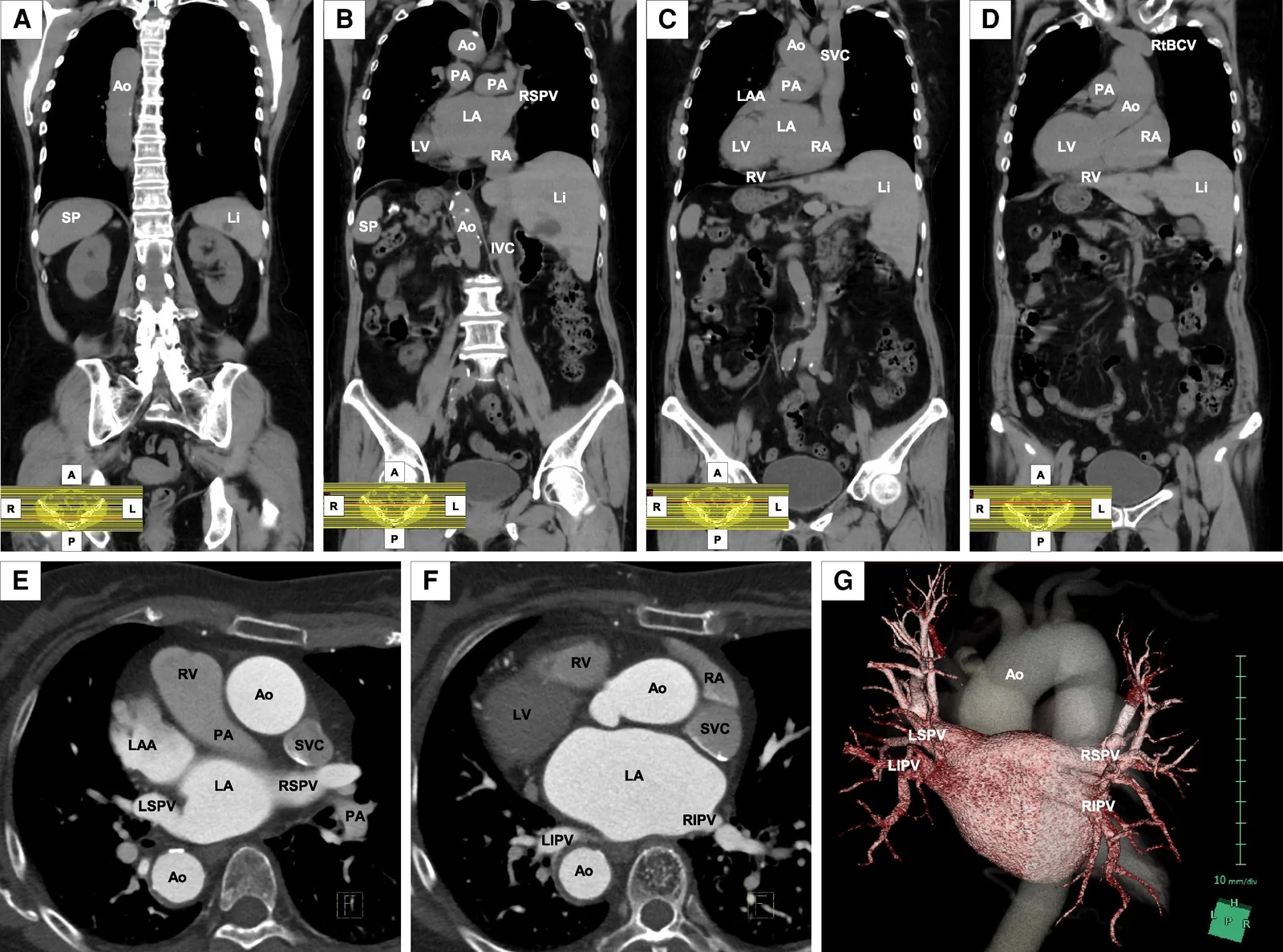
Preprocedural three-dimensional computed tomography (3D CT) demonstrating complete situs inversus with dextrocardia (SID) without additional anatomic anomalies. (*A–D*) Coronal CT images showing complete mirror-image anatomy of the thoracic and upper abdominal organs. The liver (Li) is located on the left and the spleen (SP) on the right. Cardiac chambers are reversed, with the right atrium (RA) and right ventricle (RV) positioned on the left. Major thoracic vessels, including the aorta (Ao), pulmonary artery (PA), inferior vena cava (IVC), superior vena cava (SVC), and right brachiocephalic vein (RtBCV), are labelled. (*E*, *F*) Axial CT images showing the left atrium (LA), left atrial appendage (LAA), and pulmonary veins (LSPV, LIPV, RSPV, RIPV) in mirror-image configuration. (*G*) Three-dimensional reconstruction illustrating the spatial relationships between the LA and pulmonary veins in SID. Abbreviations: LIPV, left inferior pulmonary vein; LSPV, left superior pulmonary vein; LV, left ventricle; RA, right atrium; RIPV, right inferior pulmonary vein; RSPV, right superior pulmonary vein; RV, right ventricle.

A 69-year-old woman with SID was referred for CA because of drug-refractory persistent AF with associated palpitations lasting several months. Her height, weight, and body mass index were 157.0 cm, 60.8 kg, and 24.7 kg/m^2^, respectively. Laboratory data showed a brain natriuretic peptide level of 171.8 pg/mL, while haemoglobin (15.0 g/dL), serum creatinine (0.71 mg/dL), potassium (4.0 mmol/L), and thyroid-stimulating hormone (2.27 μIU/mL) were within normal ranges. The electrocardiogram showed AF with a moderate ventricular response (98 bpm) (*Figure 1A* and *B*). Chest radiography revealed dextrocardia, a gastric bubble beneath the right hemidiaphragm, and a cardiothoracic ratio of 53.6% (*Figure 1C*). Transthoracic echocardiography demonstrated normal left ventricular systolic function (ejection fraction, 62%), left atrial enlargement (56 mm), and no other structural abnormalities. Preprocedural three-dimensional computed tomography (3D CT) confirmed complete SID without any additional anatomical anomalies (*Figure 2*). After obtaining written informed consent, CA was performed under moderate sedation using a CB system. To compensate for mirrored anatomy, fluoroscopy was displayed in a mirror-image orientation, and catheter manipulation was intentionally reversed (Summary figure).^3^ Venous access was obtained via the left femoral vein, and an intracardiac defibrillation catheter was placed in the coronary sinus via the right subclavian vein. Intravenous heparin (100 IU/kg) was administered, and a double transseptal puncture was performed under fluoroscopic and intracardiac echocardiographic guidance. Activated clotting time was maintained at ≥300 s.

**Figure 3:**
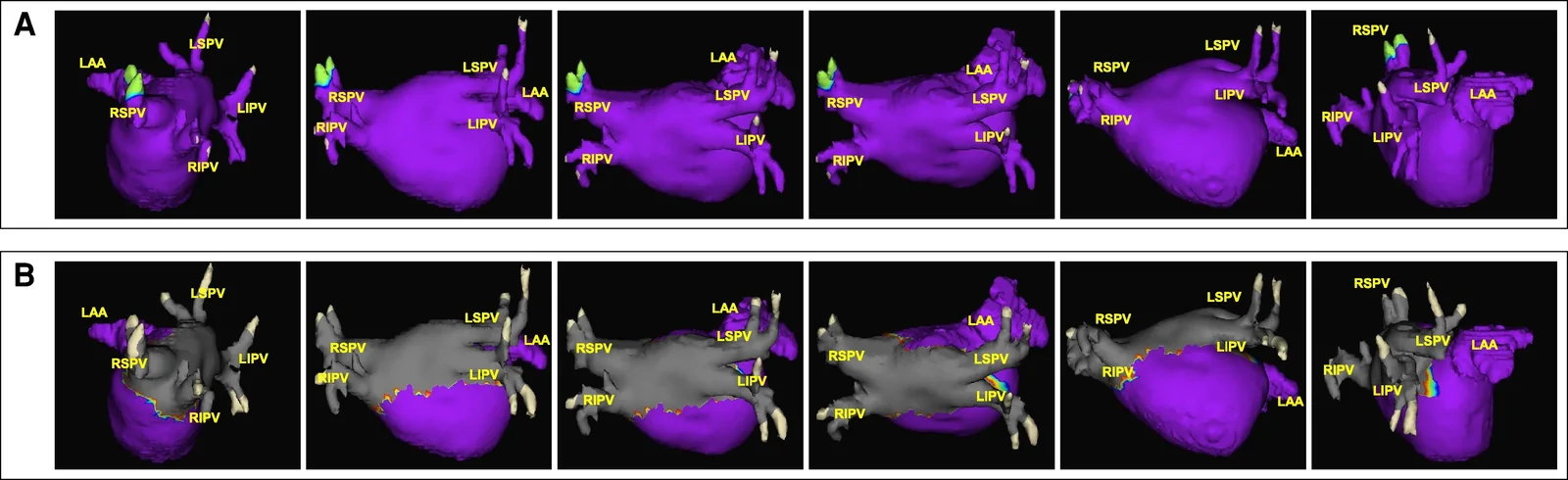
Voltage maps obtained before ablation (*A*) and after PVI + LAPWI (*B*) using a three-dimensional mapping system (EnSite velocity) and the Advisor VL circular mapping catheter. Voltage settings were 0.2–0.5 mV. The purple region represents voltages >0.5 mV, and the grey region represents voltages <0.2 mV. Abbreviations: LAA, left atrial appendage; LAPWI, left atrial posterior wall isolation; LIPV, left inferior pulmonary vein; LSPV, left superior pulmonary vein; PVI, pulmonary vein isolation; RIPV, right inferior pulmonary vein; RSPV, right superior pulmonary vein.

Baseline voltage mapping of the LAPW was performed using a circular mapping catheter (*Figure 3A*), followed by CA with a fourth-generation 28-mm CB catheter (Arctic Front Advance PRO; Medtronic). PVs were labelled according to morphological origin rather than their position in the thorax. PV isolation (PVI) was performed with 1–2 CB applications (180–240 s) per vein under a mirror-image fluoroscopic display (Summary figure). Luminal oesophageal temperature and phrenic nerve function were continuously monitored. AF persisted after PVI, and sinus rhythm was restored by internal cardioversion. PVI required five CB applications with a total ablation time of 14.0 min and a minimum temperature of −55 °C. LAPWI was subsequently performed using the previously described cross-over technique.^7^ For LA roof ablation, an inner-lumen mapping catheter (Achieve; Medtronic) was positioned deeply within the RSPV to stabilize the CB, which was advanced clockwise along the roof under anteroposterior (AP) fluoroscopy. The roof was divided into four overlapping segments (R1–R4) from left to right, and a single 120-s freeze was applied at each location (Summary figure, *B*). For LA bottom ablation, the Achieve catheter was positioned deeply in the RIPV, and the CB was similarly advanced clockwise across four segments (R1–R4) from left to right under AP fluoroscopy (Summary figure, *B*). The right pulmonary vein (RPV) carina was ablated with a single 180-s CB application by anchoring the Achieve catheter in the RSPV, deflecting the steerable sheath, and directing the CB towards the RIPV.

After the LAPW was ablated from left to right, the Achieve catheter was placed deeply in the LSPV, and the CB was advanced counterclockwise along the LA roof, divided into four overlapping segments (L1–L4) from right to left under AP fluoroscopy (Summary figure, *B*). CB ablation of the LA bottom and the left pulmonary vein (LPV) carina were performed using the same left-sided approach, with the Achieve catheter positioned in the inferior and superior PVs, respectively; this approach corresponds to the cross-over technique previously described by our group (Summary figure, *B*).^7^

**Figure 4:**
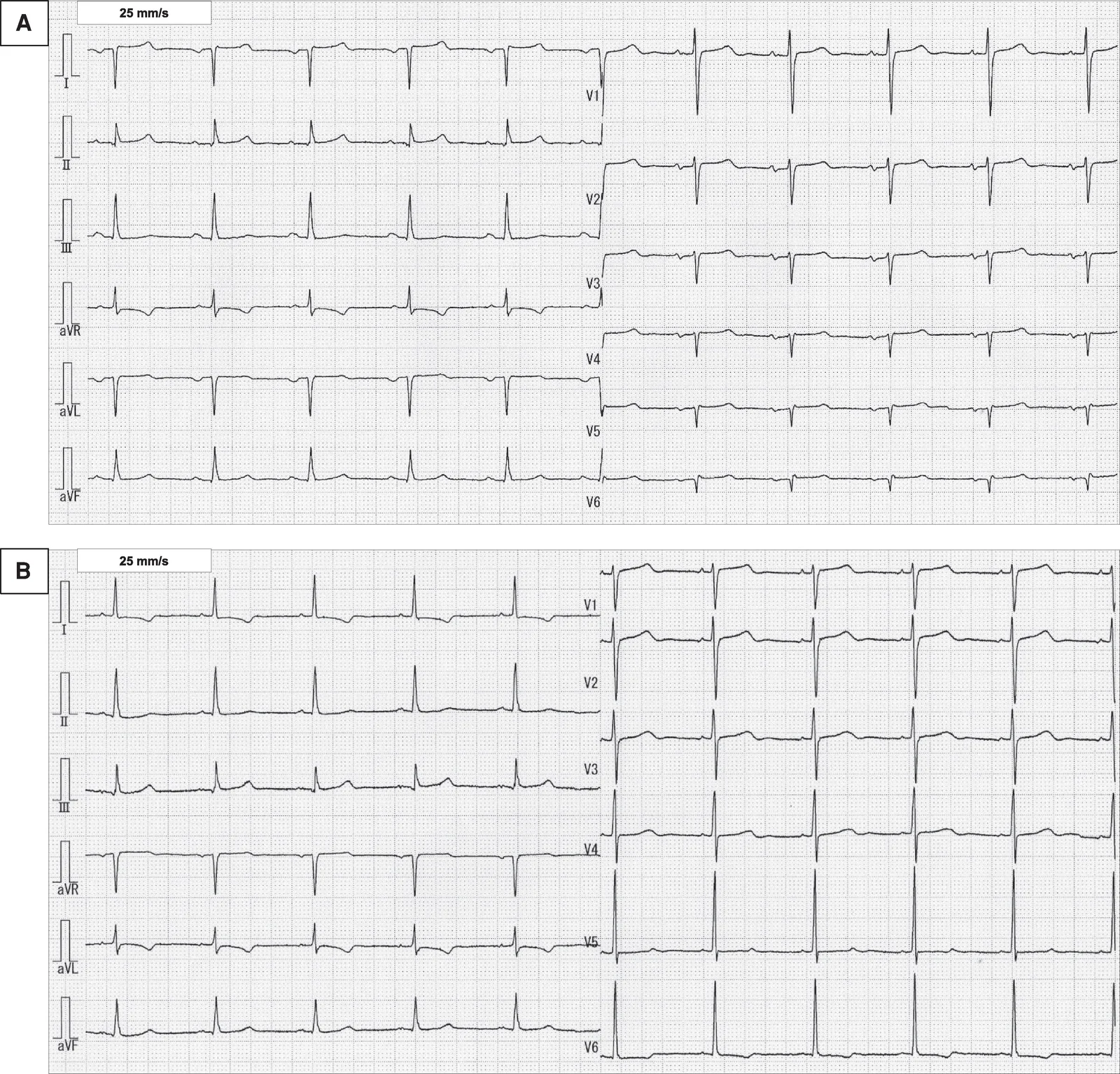
Postprocedural electrocardiogram (ECG) demonstrating sinus rhythm. (*A*) ECG recorded with standard limb and precordial lead placement, showing sinus rhythm at 63 bpm. (*B*) ECG recorded with reversed limb leads and mirror-image precordial lead placement, which provides a more conventional ECG morphology and facilitates interpretation in dextrocardia.

Complete LAPWI was confirmed by voltage mapping during left atrial appendage pacing (*Figure 3B*) and high-output pacing (10 V), demonstrating both entrance and exit block. A total of 24 CB applications were delivered for combined PVI and LAPWI. Mean minimum CB temperatures were −44.4 ± 3.4 °C for roof ablation and −38.6 ± 1.9 °C for bottom ablation. Fluoroscopy and procedure times were 70.1 and 215.0 min, respectively. No periprocedural complications occurred. The patient remained free of AF recurrence during 48 months of follow-up without antiarrhythmic therapy (*Figure 4A* and *B*).

## Discussion

SID presents unique technical challenges for AF ablation because mirrored atrial anatomy, PV orientation, and septal geometry can render standard catheter manoeuvres counterintuitive. Without deliberate procedural adaptation, the risk of spatial error during transseptal puncture and posterior wall ablation may increase.

Previous reports have demonstrated the feasibility of AF ablation in SID using radiofrequency, hot-balloon, CB, and pulsed field ablation (PFA).^2–6^ Although CB-based LAPWI in a patient with SID has recently been reported,^5^ adjunctive RFCA was required to complete left atrial bottom ablation. In contrast, the present case achieved complete LAPWI exclusively using CB applications with the cross-over technique, without adjunctive radiofrequency energy.

A key procedural strategy in this case was the use of mirror-image fluoroscopy combined with intentionally reversed catheter manipulation, which restored conventional left–right spatial perception for the operator.^3^ This adaptation facilitated safer navigation in mirrored anatomy, particularly during transseptal access and posterior wall lesion delivery.

According to the 2024 ESC Guidelines for the management of AF, CA is an established rhythm-control strategy for symptomatic AF, including selected patients with persistent AF.^8^ In the present case, persistent AF and LA enlargement supported a substrate-based ablation strategy including adjunctive LAPWI. The LAPW is increasingly recognized as an important arrhythmogenic substrate in AF and a potential adjunctive ablation target beyond PVI.^9^ Although CBA has been applied to achieve PVI with concomitant LAPWI, performing complete LAPWI using CBA alone remains technically challenging, with reported acute success rates ranging from 54% to 84%.^10^ The cross-over technique, in which overlapping CB applications are delivered from both left- and right-sided PV anchor positions, allows balloon contact from opposing orientations and may improve lesion contiguity across the posterior wall.^7^ In the present case, this approach enabled complete LAPWI despite the complex mirrored anatomy.

While PFA has gained attention for its favourable safety profile,^6,11,12^ CBA remains a widely available modality with established lesion durability. In anatomically complex settings such as SID, optimized cryoballoon strategies—including the cross-over technique—may facilitate achieving LAPWI in selected cases where lesion continuity is prioritized.

The patient remained free of AF recurrence for 48 months following combined PVI and LAPWI using CB. Compared with our previously reported CB-based LAPWI cohort involving patients with normal cardiac anatomy,^7^ the present case showed slightly longer procedure and fluoroscopy times (procedure time: 215.0 min vs. 180.0 ± 42.4 min; fluoroscopy time: 70.1 min vs. 53.1 ± 17.4 min). However, considering the mirror-image anatomy and technical challenges associated with catheter manipulation in SID, the procedure duration remained clinically acceptable. No periprocedural complications occurred, and voltage mapping confirmed complete LAPWI.

Several limitations should be acknowledged. First, this report describes a single-case experience, and the generalizability of this strategy to other patients with SID remains uncertain. Second, rhythm follow-up was based on serial outpatient electrocardiography and periodic Holter monitoring rather than continuous monitoring with an implantable loop recorder, which may have underestimated asymptomatic AF recurrence. Third, although luminal oesophageal temperature was continuously monitored throughout the procedure, the anatomical relationship between the oesophagus and the LAPW in situs inversus may differ from that in normal anatomy and could potentially influence procedural safety.

Overall, this case provides practical technical insights into performing CB-based LAPWI in anatomically mirrored hearts. Complete LAPWI using the cross-over technique was successfully achieved without adjunctive RFCA. The successful use of mirror-image fluoroscopy and intentionally reversed catheter manipulation underscores the importance of deliberate spatial adaptation when undertaking complex left atrial ablation in patients with SID.

## Lead author biography


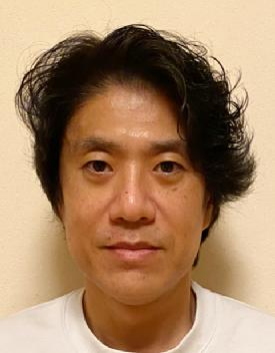


Dr. Fuminori Odagiri is a cardiac electrophysiologist at Juntendo University Urayasu Hospital, Japan. His clinical and research interests focus on catheter ablation for atrial fibrillation, including cryoballoon-based strategies, posterior wall isolation, and ablation in complex cardiac anatomy. He is also actively engaged in the evaluation and clinical implementation of emerging ablation technologies, with the aim of improving procedural safety, lesion durability, and long-term outcomes.

## Data Availability

The data underlying this article will be shared on reasonable request to the corresponding author.
